# Enumeration of Chemoorganotrophic Carbonyl Sulfide (COS)-degrading Microorganisms by the Most Probable Number Method

**DOI:** 10.1264/jsme2.ME19139

**Published:** 2020-04-29

**Authors:** Hiromi Kato, Takahiro Ogawa, Hiroyuki Ohta, Yoko Katayama

**Affiliations:** 1 Graduate School of Life Sciences, Tohoku University, 2–1–1 Katahira, Sendai 980–8577, Japan; 2 Graduate School of Agriculture, Tokyo University of Agriculture and Technology, 3–5–8 Saiwai-cho, Fuchu, Tokyo 183–8509, Japan; 3 Present address: Department of Biotechnology and Life Science, Tokyo University of Agriculture and Technology, 2–24–16 Nakamachi, Koganei, Tokyo, 184–8588, Japan; 4 Department of Bioresource Science, Ibaraki University College of Agriculture, 3–21–1 Chuou, Ami-machi, Ibaraki 300–0393, Japan; 5 Independent Administrative Institution, Tokyo National Research Institute for Cultural Properties, 13–43 Ueno Park, Taito-ku, Tokyo 110–8713, Japan

**Keywords:** carbonyl sulfide, soil microbes, most probable number method, carbonic anhydrase

## Abstract

Carbonyl sulfide (COS) is the most abundant sulfur compound in the atmosphere, and, thus, is important in the global sulfur cycle. Soil is a major sink of atmospheric COS and the numerical distribution of soil microorganisms that degrade COS is indispensable for estimating the COS-degrading potential of soil. However, difficulties are associated with counting COS-degrading microorganisms using culture-dependent approaches, such as the most probable number (MPN) method, because of the chemical hydrolysis of COS by water. We herein developed a two-step MPN method for COS-degrading microorganisms: the first step for chemoorganotrophic growth that supported a sufficient number of cells for COS degradation in the second step. Our new MPN analysis of various environmental samples revealed that the cell density of COS-degrading microorganisms in forest soils ranged between 10^6^ and 10^8^ MPN (g dry soil)^–1^, which was markedly higher than those in volcanic deposit and water samples, and strongly correlated with the rate of COS degradation in environmental samples. Numerically dominant COS degraders that were isolated from the MPN-positive culture were related to bacteria in the orders *Bacillales* and *Actinomycetales*. The present results provide numerical evidence for the ubiquity of COS-degrading microbes in natural environments.

Carbonyl sulfide (COS, O=C=S) is an atmospheric trace gas with an average mixing ratio of approximately 500 parts per trillion by volume (pptv, pL L^–1^); however, it is the most abundant sulfur compound in the troposphere because of its long life (Chin and Davis, 1995; Montzka *et al.*, 2007). COS is a major source of the sulfate layer in the stratosphere and indirectly affects Earth’s radiation budget (Baldwin *et al.*, 1976; Crutzen, 1976; Chin and Davis, 1995; Andreae and Crutzen, 1997; Brühl *et al.*, 2012); therefore, efforts have been devoted to investigating the sinks and sources of COS and calculating the mass budget of atmospheric COS (Watts, 2000; Berry *et al.*, 2013). Vegetation and soil are the main sinks for atmospheric COS (Kuhn *et al.*, 1999; Watts, 2000; Kettle *et al.*, 2002; Berry *et al.*, 2013; Whelan *et al.*, 2018). Although the physiological mechanisms of COS degradation and estimations of the global uptake of atmospheric COS by vegetation have been examined (Whelan *et al.*, 2018), limited information is still currently available on soil microorganisms. The significance of soil as a source and sink of COS remains unclear (Whelan *et al.*, 2016; 2018) because COS exchange between soil and the atmosphere is the net result of the uptake and production of COS, and both processes are affected by a number of environmental parameters, including temperature, moisture, porosity, biomass, and microbial community (Kesselmeier *et al.*, 1999; Yi *et al.*, 2007; Van Diest and Kesselmeier, 2008; Bunk *et al.*, 2017; Behrendt *et al.*, 2019; Meredith *et al.*, 2019). Prokaryotic and fungal activities strongly contribute to COS degradation in soil (Kato *et al.*, 2008; Masaki *et al.*, 2016; Meredith *et al.*, 2019). The enzymatic mechanisms of microbial COS degradation have been examined in several bacterial groups, such as the obligate chemolithoautotrophic sulfur-oxidizing bacterium *Thiobacillus thioparus* strain THI115 (COS hydrolase/COSase) (Ogawa *et al.*, 2013), acidophilic archaea in the genus *Acidianus* (CS_2_ hydrolase) (Smeulders *et al.*, 2011), and acidophilic bacteria in the genus *Acidithiobacillus* (CS_2_ hydrolase) (Smeulders *et al.*, 2013). These enzymes belong to the beta-class carbonic anhydrase (*β*-CA) family, which catalyzes reversible hydration between CO_2_ and HCO_3_^–^ and also reacts with COS (COS+H_2_O→CO_2_+H_2_S) because of the structural analogy between CO_2_ and COS (Protoschill-Krebs and Kesselmeier, 1992; Protoschill-Krebs *et al.*, 1995; Protoschill-Krebs *et al.*, 1996; Blezinger *et al.*, 2000). These findings suggest that soil microbes possessing the *β*-CA family contribute to COS degradation *in situ* (Kesselmeier *et al.*, 1999; Ogawa *et al.*, 2016; Meredith *et al.*, 2019). In contrast to the extensive accumulation of evidence on their enzymology and genetics, the cell densities of microorganisms with the ability to degrade atmospheric COS in soil environments remain unclear. Culture-independent and -‍dependent methods are both required to obtain a more detailed understanding of the environmental distribution of COS-degrading microbes. Regarding the former method, genes encoding one clade of *β*-CA are candidate targets of quantitative PCR (Ogawa *et al.*, 2016; Meredith *et al.*, 2019); however, the sequence diversity of the *β*-CA family has hampered the design of appropriate primers that cover the regions that are important for the specificity and functionality of COS degradation (Ogawa *et al.*, 2016). Although the latter method has the advantage of directly measuring COS-degrading activity, these activity-based approaches have not yet been attempted. Therefore, the aim of the present study is to provide numerical data on viable cells that exhibit COS-degrading activity and compare them among various environments. The most probable number (MPN) method has been widely used to estimate the cell densities of the microorganisms involved in the carbon, nitrogen, and sulfur cycles (*e.g.* methanogenic/methanotrophic [Adachi, 2001], denitrifying [Braker *et al.*, 2010], and sulfur-oxidizing microbes [Li *et al.*, 2008]). However, difficulties are associated with quantifying biological COS degradation due to the chemical hydrolysis of COS (Elliott *et al.*, 1989) by water in bacterial cultures. Therefore, we herein developed a two-step MPN method for COS-degrading microorganisms: normal MPN methods were initially performed for chemoorganotrophs, and pre-grown cells were then used in the first step to assess COS-degrading activity. The cell densities of COS-degrading microbes obtained from various environments strongly correlated with the COS-degrading activities of their sources. Our two-step MPN method will facilitate estimations of the potential of soil microbes to degrade COS. Furthermore, the phylogenetic affiliations of COS-degrading isolates support the relationships between the COS-degrading activity and phylogeny of *β*-CA (Ogawa *et al.*, 2013; Ogawa *et al.*, 2016). The present results provide novel insights into the ecology and mechanisms of bacterial COS degradation in soil.

## Materials and Methods

### Environmental samples

Soil samples were collected from the surface layer (0 to 10‍ ‍cm) beneath the litter. Brown forest soil samples were obtained from Mt. Karasawa (KS-13, Field Science Center, Tokyo University of Agriculture and Technology, Tochigi, Japan) in 2006 and from Mt. Sengen-yama park (SG-1, Tokyo, Japan) in 2005 (Table 1). We also collected volcanic samples from Mt. Fuji (Shizuoka and Yamanashi, Japan) and Miyake-jima island (an island approximately 180‍ ‍km south of Tokyo) for comparisons with forest soils. Mt. Fuji is an isolated stratovolcano with a summit that is 3,776‍ ‍m above sea level (a.s.l.). Vegetation at the summit is limited to lichens and mosses at restricted areas. Soil was collected at seven sites along the mountain slope from the summit to the foot (sampling sites at 1,140 and 1,300‍ ‍m a.s.l. were located in vegetated areas) in 2007 (Kato *et al.*, 2012). On Miyake-jima island, repeated volcanic eruptions (the latest in 2000) have formed sites with different stages of plant succession. In 2006, we collected a volcanic ash layer and the underlying soil layer near the crater (OY) and in a forest damaged by volcanic gases and ash (IG), and forest soil that was unaffected by the latest eruption (CL) (Fujimura *et al.*, 2016). Scoria samples were collected from an unvegetated area (KP-1), at the edge of a vegetated area (KP-3), and within the vegetated area (KP-5) (King *et al.*, 2008). All samples were sieved through a 2-mm mesh, stored at 4°C, and analyzed for soil parameters and COS-degrading activities within one month of sampling. Water samples were collected from the surface of experimental ponds at Tokyo University of Agriculture and Technology. Ponds A and B were eutrophic, and treatments to remove phosphorus and nitrogen from pond C maintained transparency of >50‍ ‍cm. The chemical properties of all samples are summarized in Table 1.

### Enumeration of chemoorganotrophic COS degraders by the MPN method

**Fig. 1.**
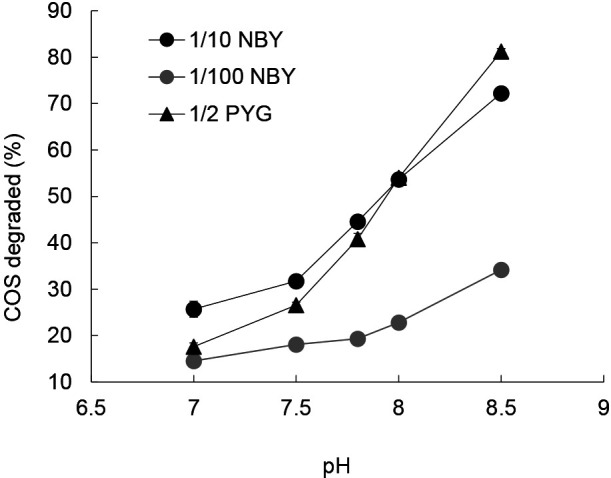
Effects of medium pH on the chemical hydrolysis of COS degradation. The degradation of COS at 30 ppmv for 24 h in sterilized 1/10 NBY (black circle), 1/2 PYG (black triangle), and 1/100 NBY (gray circle) media was quantified.

In the present study, we focused on COS degraders in chemoorganotrophs that may be cultivated in routinely used media, such as diluted NB medium. However, the growth of chemoorganotrophic microbes in our three types of media: 1/10-NBY (nutrient broth with yeast extract), 1/100-NBY, and 1/2-PYG, frequently alkalized the pH of these media. COS is relatively stable towards acids and its hydrolysis is accelerated at an alkaline pH (Ferm, 1957). The alkalization of our media enhanced the chemical hydrolysis of COS (Fig. 1). Therefore, we supplemented phosphate buffer to avoid the alkalization of media. 1/10-NBY medium contained (L^–1^) 1.0‍ ‍g of meat extract (Kyokuto Pharmaceutical Industrial), 1.0‍ ‍g of Bacto peptone (Difco), 0.5‍ ‍g of Bacto yeast extract (Difco), 0.5‍ ‍g of NaCl, and 100‍ ‍mL of 1 M phosphate buffer (pH 7.0). 1/100-NBY medium was 1/10-diluted 1/10-NBY. 1/2-PYG medium contained (L^–1^) 1.0‍ ‍g of polypeptone (Nihon Seiyaku), 0.5‍ ‍g of Bacto yeast extract, 0.25‍ ‍g of glucose, and 100‍ ‍mL of 1 M phosphate buffer (pH 7.0).

Three grams of environmental samples was added to 27‍ ‍mL of sterilized distilled water in a 50-mL vial and then suspended by sonication (UT-205S; Sharp) for 5‍ ‍min. One milliliter of ten-fold serial dilutions of the suspension was inoculated into 9‍ ‍mL of medium in a glass tube (1.5‍ ‍cm i.d.×18‍ ‍cm length), and triplicate cultures of each dilution level were incubated at 30°C in the dark with reciprocal shaking (120 rpm). The MPN method for chemoorganotrophs was performed based on increases in turbidity (first step). To measure the COS-degrading activity of the culture, 2‍ ‍mL of each culture on days 4, 11, 18, and 40 of the first step was transferred into a sterilized glass test tube (1.2‍ ‍cm i.d.×15‍ ‍cm length), which was then tightly sealed with a butyl rubber cap. COS standard gas (10% in N_2_; Nissan Tanaka) was added to give a final concentration of 30 parts per million by volume (ppmv, μL L^–1^), and the test tubes were then incubated as described above. Twenty-four hours after the addition of COS, residual COS was quantified by gas chromatography (see below), and microbial COS degradation was assessed by comparisons to chemical COS degradation in a control test tube containing sterilized medium and COS (second step): a degradation ratio of ≥40% indicated the marked microbial degradation of COS (see Results). Numbers in positive cultures for COS degradation were counted at each dilution level and used to give the cell density (MPN [g dry soil]^–1^) of COS-degrading microbes estimated based on the three-tube MPN table (Oblinger and Koburger, 1975).

### Phylogenetic analysis of COS-degrading bacteria

To examine the phylogeny of COS-degrading bacteria in soil samples, we isolated bacteria from cultures that exhibited COS-degrading activity. Small parts of the cultures at the highest and second highest dilution levels that showed positive reactions in the medium used were spread onto plates of 1/10-NBY medium supplemented with 15‍ ‍g L^–1^ agar and incubated at 30°C for 10‍ ‍d in the dark. A pure isolated bacterium was obtained by the repeated spreading out of a single colony. Each isolate was grown in 10‍ ‍mL of medium, and 2‍ ‍mL of the culture at an early stationary phase was used to quantify COS degradation as described above.

Cultures of isolates were centrifuged at 15,000×*g* for 15‍ ‍min and cell pellets were used to extract genomic DNA using the Nexttec Genomic DNA Isolation Kit for Bacteria (Nexttec GmbH). The V1–V4 regions of the 16S rRNA gene were amplified by PCR using the eubacterial universal primers 27F (5′-GAGTTTGATCCTGGCTCAG-3′) and 907R (5′-CCCCGTCAATTCCTTTGAGTTT-3′) (Weisburg *et al.*, 1991; Muyzer *et al.*, 1993). The PCR mixture contained PCR buffer with 1.5‍ ‍mM Mg^2+^ (Qiagen), 0.2‍ ‍mM dNTPs, 0.1‍ ‍μM of each primer, 1.25 units Hotstar Taq Plus DNA polymerase (Qiagen), and 0.1–1‍ ‍ng template DNA. Amplification was performed at an initial 95°C for 5‍ ‍min; 10 cycles of 94°C for 1‍ ‍min, 65–56°C (decreased by 1°C per cycle) for 1‍ ‍min, and 72°C for 1‍ ‍min; 20 cycles of 94°C for 1‍ ‍min, 55°C for 1‍ ‍min, and 72°C for 1‍ ‍min; and a final 72°C for 10‍ ‍min. The PCR product was sequenced with the BigDye Terminator Cycle Sequencing Kit (Applied Biosystems) and a 3130/3130xl Genetic Analyzer (Applied Biosystems). 16S rRNA gene sequences were compared with sequences in the Ribosomal Database Project, and taxonomically assigned by the RDP classifier (Wang *et al.*, 2007).

### Rate constant of COS degradation by environmental samples or isolated bacteria

To compare the rates of COS degradation among environmental samples, we quantified the rate constant of COS degradation (Kato *et al.*, 2008). Four grams (wet weight) of a bulk soil sample or 10‍ ‍mL of a water sample was added to a glass test tube (2‍ ‍cm i.d.×20‍ ‍cm length), which was then sealed with a butyl rubber cap, followed by the addition of COS gas to a final concentration of 30‍ ‍ppmv. The time courses of COS degradation were fit to the exponential function *C*(*t*)=*C_0_*·e^–k^*^t^*, where *C*(*t*) is the concentration of COS at time *t* (h), *C*_0_ is the initial COS concentration, and k is the rate constant (h^–1^ test tube^–1^).

The rate constants of COS degradation by bacterial cultures were assessed based on the same procedure as that described above. However, due to differences in cell growth among the isolates, particularly in the case of *Streptomyces* (see below), difficulties were associated with comparing the results obtained. Therefore, we measured biomass carbon in each culture and used these values to normalize rate constants. Ten milliliters of the bacterial culture at the early stationary phase in 1/10 NBY or PYG medium was centrifuged at 6,000×*g* for 15‍ ‍min and washed twice with phosphate-buffered saline (PBS), and cells were then re-suspended in 10‍ ‍mL of PBS. The resultant suspension was subjected to the quantification of rate constants and biomass. We assayed the biomass of each cell suspension using a wet-oxidation non-dispersive infrared gas analyzer (Seto and Tange, 1980). Organic carbon derived from the biomass was oxidized to CO_2_ by catalytic silver nitrate and potassium persulfate under boiling conditions, and CO_2_ was injected into a non-dispersive infrared gas analyzer (ZRH; Fuji Electric) with CO_2_-free air as the carrier gas. The quantity of CO_2_ was compared against a standard curve obtained from 0.05, 0.5, 1, and 3‍ ‍mg carbon of glucose to calculate total organic carbon. The rate constant of each cell suspension, minus that of an uninoculated blank, was divided by its biomass carbon. We compared this specific rate constant normalized by biomass (SRCB) among the isolates. Unless otherwise indicated, measurements were performed in three biological replicates.

*Mycobacterium* sp. strain THI401 was also examined on the rate constant for comparisons because this bacterium was isolated from forest soil and exhibited strong COS-degrading activity at a wide range of COS concentrations between 500 pptv (atmospheric level) and 2,000 ppmv (Kato *et al.*, 2008).

### COS analysis

COS concentrations were measured using a gas chromatograph (GC-14B; Shimadzu) equipped with a flame photometric detector and glass column packed with Porapak QS (50–80 mesh; Waters Associates) as described previously (Katayama *et al.*, 1992). Nitrogen gas was used as the carrier and the flow rate was 43‍ ‍mL min^–1^. The temperatures of the injector, column, and detector were 150, 110, and 150°C, respectively. The detection limit of COS with a direct injection into the gas chromatograph was 1.97 ppmv.

### Nucleotide sequence accession numbers

The nucleotide sequences of the 16S rRNA genes of bacteria isolated in the present study have been deposited in the DNA Data Bank of Japan under the accession numbers LC099885–LC099924.

## Results

### Rate constants of the COS degradation of environmental samples

We collected environmental samples that had rate constants of COS degradation ranging between 0.01 and 1.71‍ ‍h^–1^ (g dry soil)^–1^ (Table 1): forest soil had higher rate constants than scoria and pond water. COS degradation by soil depends on temperature (Kesselmeier *et al.*, 1999). Temperatures at the summit of Mt. Fuji during the sampling periods ranged between –3 to 7°C. Regarding the adaptation of microbes at the summit to very low temperature, we tested scoria samples at 4°C. The rate constant at 4°C was 0.094 h^–1^ (g dry soil)^–1^, and did not exceed the value of 0.26‍ ‍h^–1^ (g dry soil)^–1^ obtained at 30°C. We used the rate constants obtained at 30°C in further comparisons for simplicity.

### Development of the MPN method for enumerating COS-degrading microbes

The chemical hydrolysis of COS by water makes it difficult to differentiate not only biological COS degradation from chemical hydrolysis, but also possible microbial growth using H_2_S as an energy source that was derived from the chemical hydrolysis of COS. We hypothesized that microbial COS degradation may be evaluated in the short-term by using cultures that have grown under chemolithoautotrophic or chemoorganotrophic conditions. However, cultures grown under chemolithoautotrophic conditions did not exhibit significant levels of COS degradation, as described below. Therefore, we herein focused on the construction of the MPN method to count chemoorganotrophic COS-degrading microbes in environmental samples.

We initially differentiated microbial COS degradation from chemical hydrolysis using forest soil collected at Mt. Karasawa (KS-13). Microbial COS degradation was examined over 24 h using cells that grew in the three types of liquid media used, namely, 1/10 NBY, 1/100 NBY, and 1/2 PYG. Most of the cultures in 1/10 NBY medium degraded >40% of the initial 30 ppmv COS in 24 h (Table 2), which was greater than the chemical hydrolysis of COS (from 26% at pH 7.0 to 32% at pH 7.5, Fig. 1). Since the pH of all cultures was maintained at less than pH 7.5 (mainly less than pH 7.3) during the incubation, we concluded that a degradation ratio of ≥40% indicated a strong microbial contribution to COS degradation. It is important to note that several cultures at higher dilution levels (*e.g.* culture No. 8-2, Table 2) transiently exceeded the ratio of 40%, indicating the need for repeated measurements of COS-degrading activities during the first step in order to avoid underestimations of the number of positive cultures. Based on the number of cultures showing biological COS degradation, the cell density of COS-degrading microbes in KS-13 was estimated to be 9.6×10^8^ MPN (g dry soil)^–1^, which was similar to that of chemoorganotrophic microbes in the sample. Lower cell densities of COS-degrading microbes were detected in 1/2-PYG medium at 9.6×10^7^ MPN (g dry soil)^–1^ and in 1/100-NBY medium at 2.7×10^4^ MPN (g dry soil)^–1^ (Table S1). The markedly lower cell density in 1/100-NBY medium may have been due to the lower carbon content of this medium than those of 1/10-NBY and 1/2-PYG. The MPN method for COS degraders strongly depends on the biomass of the culture, as described below. Therefore, the initial carbon contents and incubation period in the first step were important factors influencing COS-degrading activity. In subsequent experiments, we used 1/10-NBY in the first step of MPN and then measured COS-degrading activity not once but three times using the culture sampled on days 4, 11, and 20 of the first step.

### Abundance of COS-degrading microbes in soil and water

**Fig. 2.**
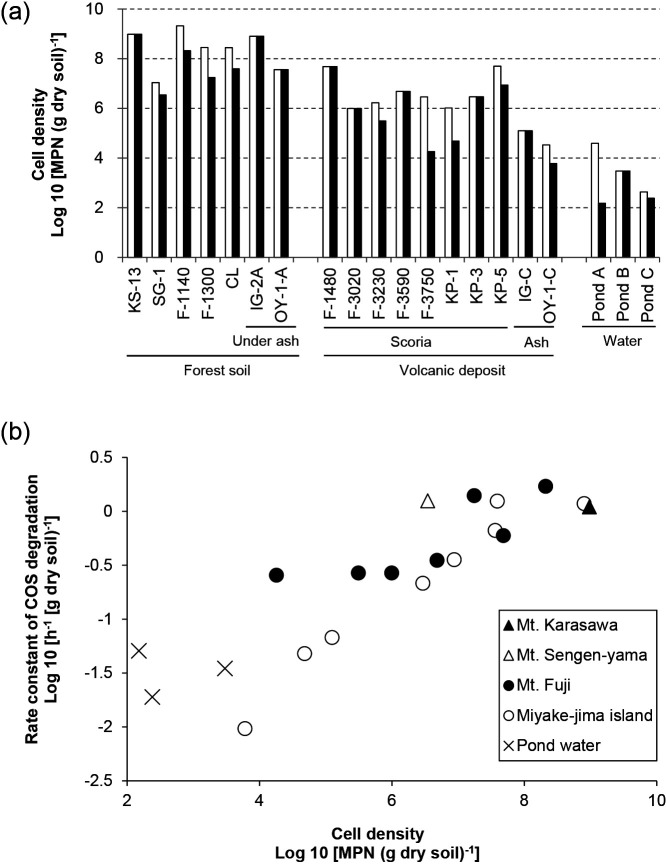
Cell density of chemoorganotrophs and COS-degrading microbes in soil and water samples. (a) MPN counting for chemoorganotrophs (white bar) and COS-degrading microbes (black bar) was based on the turbidity of MPN cultures and triplicate assays of COS-degrading activity (the case of Mt. Karasawa was shown in Table 2), respectively. (b) Relationships between the cell density of COS-degrading microbes and the rate constant of COS degradation by environmental samples. The correlation coefficient between the logarithmic values of COS degraders and those of the rate constants was 0.881 (*P*<0.01).

By using the constructed MPN method, we counted COS-degrading microbes in other environmental samples of forest soil, volcanic deposits, and eutrophic pond water. COS degraders in these samples were distributed at densities of 10^2^ to 10^8^ MPN h^–1^ (g dry soil)^–1^ or mL^–1^. Higher densities of COS degraders were found in forest soils that also showed higher densities of chemoorganotrophs (*e.g.*, KS-13, SG-1, F-1300, F-1140, CL, IG-2A, and OY-1-A; Fig. 2a). Lower densities of COS degraders and chemoorganotrophs were detected in scoria and pond water samples. In 16 out of 20 samples, COS degraders accounted for >10% of chemoorganotrophs (Fig. 2a). The values of the rate constant of COS degradation in environmental samples appeared to be a power function of the MPN values of COS-degrading microbes (*y*=0.0043 *x*^0.2941^, *r*^2^=0.7765), and linear relationships among logarithms of rate constants and MPN values showed strong correlations (Pearson’s correlation coefficient=0.881, *P*<0.01; Fig. 2b).

### Phylogenetic distribution of COS-degrading bacteria

**Fig. 3.**
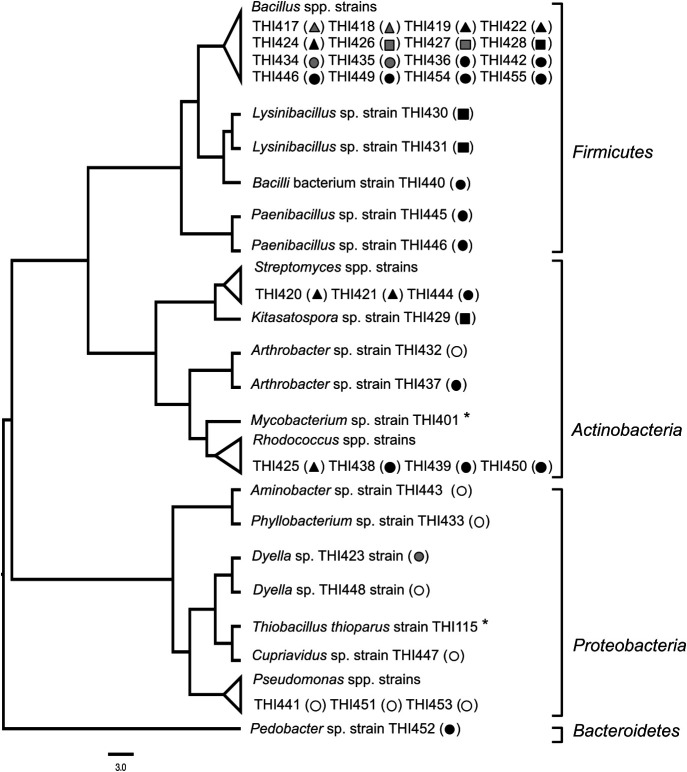
Phylogenetic tree of 16S rRNA genes of COS-degrading bacteria and COS non-degrading bacteria isolated from MPN cultures of Mt. Karasawa (triangle), Mt Fuji (circle), and Miyake-jima island (square). The COS-degrading activities of isolates are indicated by a color index: less than 40% (white), 40 to 60% (gray), and more than 60% (black) of initial COS of 30 ppmv were degraded in a 24-h incubation. The tree was constructed using the neighbor-joining method (Saitou and Nei, 1987). COS-degrading bacteria from previous studies (Kato *et al.*, 2008; Ogawa *et al.*, 2013) are indicated by asterisks.

We isolated 32 strains of COS-degrading bacteria from the MPN cultures of soil samples that degraded >40% of the initial COS in 24 h, and eight COS non-degrading isolates. 16S rRNA gene sequencing revealed that, regardless of the source for sampling, most COS-degrading isolates were affiliated into two orders: *Bacillales* involving *Bacillus*, *Lysinibacillus*, and *Paenibacillus*, and *Actinomycetales* involving *Streptomyces*, *Kitasatospora*, and *Rhodococcus* (Fig. 3, black and gray symbols). On the other hand, most of the COS non-degrading isolates were affiliated into *Proteobacteria* (Fig. 3, white symbols). COS-degrading and COS non-degrading isolates were found in the genus *Dyella* in *Gammaproteobacteria* and *Arthrobacter* in *Actinobacteria*. Thus, most of the culturable and numerically dominant COS degraders isolated from soil samples belonged to the orders *Bacillales* and *Actinomycetales*.

### Comparison of “SRCB” of COS degradation among bacterial isolates

**Fig. 4.**
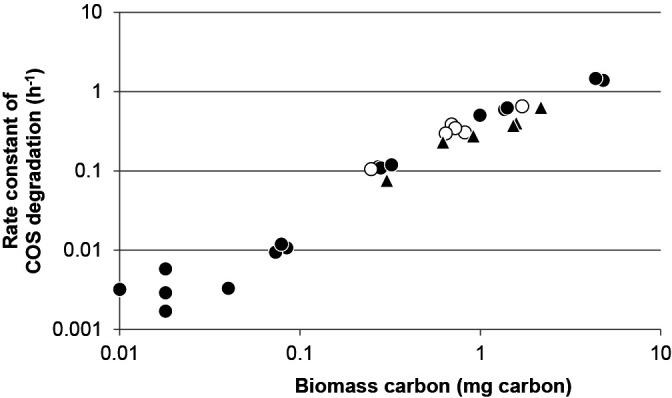
Effects of the biomass of a cell suspension on the rate constant of COS degradation. Different amounts of the biomass of a cell suspension (x-axis) were used to assess the rate constant of COS degradation (y-axis) by bacterial strains of *Bacillus* sp. strain THI418 (closed circle), *Streptomyces* sp. strain THI420 (open circle), and *Streptomyces* sp. strain THI421 (closed triangle).

To compare COS-degrading activities among isolates, we examined the effects of the biomass on the rate constant of COS degradation. When the biomass of the cell suspension was in the range of 0.2 to 5‍ ‍mg, the rate constant was linearly dependent on the biomass (Pearson’s correlation coefficient=0.98, *P*<0.01; Fig. 4). Therefore, microbial COS degradation depended on both the concentration of COS and biomass carbon of the cell suspension; rate constants may be compared when normalized to the biomass as SRCB at least in this range (Seto, 1986). We assessed the SRCB of COS-degrading bacteria isolated from forest soil at Mt. Karasawa, at which the highest MPN was obtained, and from volcanic samples on Miyake-jima island (Table 3). *Bacillus* spp. strains THI419, THI424, THI426, THI427, and THI428 and *Kitasatospora* sp. strain THI429 exhibited high SRCB values (>1.0 h^–1^ mg^–1^ carbon), which were similar to or higher than the value for *Mycobacterium* sp. strain THI401 (2.6 h^–1^ mg^–1^ carbon). Although the number of tested strains was small, high SRCB values were more frequently observed in isolates from forest soil and volcanic samples from Miyake-jima island than in those from forest soil from Mt. Karasawa.

## Discussion

Sulfide is the most reduced species among sulfur compounds, and plays the following vital roles in organisms: as an electron donor for sulfur-oxidizing microorganisms, in the biosynthesis of sulfur-containing amino acids (Kertesz, 2000), as a signal molecule (Luhachack and Nudler, 2014), and as an antioxidant agent (Shatalin *et al.*, 2011). A more detailed understanding of the biological importance of sulfide has accelerated the study of sulfide synthesis from sulfate, sulfonates, and sulfate esters (Kertesz, 2000). The microbial reaction that generates H_2_S from atmospheric sulfur compounds, such as COS and CS_2_, was recently detected in prokaryotes (Smeulders *et al.*, 2011; Ogawa *et al.*, 2013); however, the environmental distribution of microorganisms harboring these metabolic pathways remains unknown. In the present study, we developed a new MPN method to quantify chemoorganotrophic bacteria harboring COS-degrading activity. We initially attempted to count them under chemolithoautotrophic conditions, which was achieved using media for sulfur-oxidizing bacteria. The forest soil sample SG-1 was subjected to the MPN analysis using minimal basal medium (Li *et al.*, 2008) supplemented with 20‍ ‍mM thiosulfate as the sole energy source (first step), and then to measurements of COS-degrading activity in the same manner to the MPN for chemoorganotrophs (second step). The MPN value of sulfur-oxidizing bacteria in the forest soil sample from Mt. Sengen-yama park was 3.5×10^5^ MPN (g dry soil)^–1^; however, these MPN cultures did not show significant COS degradation (Table S1). Therefore, alterations in the compositions of media that are appropriate for counting chemolithoautotrophic COS-degrading microbes are needed.

We applied the MPN method to enumerate chemoorganotrophic COS degraders colonizing various environmental samples, and found that forest soil samples, which exhibited stronger activity for COS degradation than other samples used in the present study, housed COS-degrading microbes at densities ranging between 10^6^ and 10^8^ MPN (g dry soil)^–1^. The high densities of COS-degrading microbes found in the present study may contribute to COS degradation by soil because MPN values strongly correlated with the rate constants of COS degradation by soils (Fig. 2). These results are useful for understanding the ecological distribution of COS-degrading microorganisms and estimating the potential of soil to degrade COS. The high MPN values observed in forest soils are consistent with previous findings reported by Bunk *et al.* (2018) showing larger COS uptake rates in forest soils than in other soils (Bunk *et al.*, 2018). In most samples, the MPN values of COS degraders were similar to or slightly lower than those of chemoorganotrophs, indicating that high percentages of culturable and numerically dominant chemoorganotrophs in soils degrade COS. COS degradation by soil samples roughly depends on the biomass (Yi *et al.*, 2007), and soils do not need to acclimatize to COS in order to uptake COS irrespective of its concentration (Saito *et al.*, 2002). The high frequency of COS degraders among chemoorganotrophs in our samples may be one reason why soils degrade COS without acclimation. The sampling sites OY and IG in Miyake-jima island have been exposed to volcanic gases, such as SO_2_ (Fujimura *et al.*, 2016) and possibly COS (Belviso *et al.*, 1986), and, thus, isolates from these sites were more likely to have high SRCB values (Table 3). On the other hand, the rate constant of COS degradation of these volcanic samples was lower than those of the forest samples (Table 1). Volcanic ash samples from Miyake-jima island and scoria samples from Mt. Fuji contained low levels of total organic carbon (Table 1), which may not support a sufficient abundance of chemoorganotrophs for strong COS-degrading activities. In contrast to light-dependent atmospheric COS uptake by plants, COS degradation by soil microbes is light-independent, and, thus, particularly important for the nighttime uptake of COS (Commane *et al.*, 2015). Further information on the relationships between soil types and COS degradation rates is needed to more accurately estimate the contribution of soil to the global budget of atmospheric COS (Watts, 2000; Berry *et al.*, 2013). To the best of our knowledge, soils showing COS degradation rate constants higher than 1.0 h^–1^ (g dry soil)^–1^ harbored chemoorganotrophic COS degraders with cell densities higher than 10^6^ MPN (g dry soil)^–1^.

The majority of the COS-degrading bacteria obtained in‍ ‍the present study belonged to the orders *Bacillales* and‍ ‍*Actinomycetales*. Previously isolated COS-degrading prokaryotes included thiocyanate-degrading *T. thioparus* (Ogawa *et al.*, 2013), CS_2_-degrading *Paracoccus denitrificans* (Jordan *et al.*, 1997), *Acidianus* sp. (Smeulders *et al.*, 2011), and *Acidithiobacillus thiooxidans* (Smeulders *et al.*, 2013) as well as anaerobic CO-utilizing bacteria (Smith *et al.*, 1991). The present results indicate that a number of well-known bacterial groups in soils exhibit COS-degrading activity. The five strains of *Bacillus* and one strain of *Kitasatospora* had SRCB values that were higher than or similar to that of *Mycobacterium* sp. THI401, which is a soil bacterium that exhibits strong COS-degrading activity (Kato *et al.*, 2008). We previously reported that strains with a SRCB value higher than 1.0 h^–1^ mg^–1^ carbon exhibited the ability to degrade atmospheric COS (Kato *et al.*, 2008; Ogawa *et al.*, 2016). These findings imply that some bacteria in the genera *Bacillus* and *Kitasatospora* play a role in the degradation of atmospheric COS. It is important to note that the phylogenetic affiliation of COS-degrading isolates observed in the present study corresponded to bacteria carrying clade D of *β*-CA (D-CA), which consists of *β*-CAs from *Actinomycetales*, *Firmicutes*, and *Archaea*, as well as COSase and CS_2_ hydrolase, and enzymes in this clade may be ancient and specialized for COS rather than for CO_2_ (Smith *et al.*, 1999; Smith and Ferry, 2000; Smeulders *et al.*, 2011; Ogawa *et al.*, 2013). This phylogeny prompted us to speculate that COS-degrading soil bacteria harbor genes that encode D-CA. We previously reported that phylogenetically diverse *Actinomycetales* from culture collections exhibited COS-degrading activity and that they possessed genes for D-CA (Ogawa *et al.*, 2016). Furthermore, a metatranscriptome analysis of soils revealed that COS consumption by soil was strongly related to the gene expression of *β*-CA, particularly D-CA (Meredith *et al.*, 2019). Our results, which were obtained using the activity-based MPN approach, provide addition support for the relationships between the phylogeny of *β*-CA and COS degradation in natural environments. Nevertheless, we cannot deny the possibility that there was a bias in the media used for MPN, which may have influenced the culturability of COS-degrading microbes. Recent studies demonstrated the fungal contribution to COS uptake in soil environments (Masaki *et al.*, 2016; Behrendt *et al.*, 2019). It is important to note that we observed aggregates of fungal hyphae in several MPN cultures from soil samples; however, due to the conditions of our media, we were unable to distinguish the fungal activity of COS degradation from bacterial activity. The use of antibiotics in MPN may be helpful for selectively counting bacterial or fungal COS degraders in environmental samples. Furthermore, more than 99% of soil microbes are difficult to cultivate under laboratory conditions. Our preliminary survey for D-CA in the GenBank database (https://www.ncbi.nlm.nih.gov), based on a BlastP analysis of D-CA and COS-degrading enzymes against available genomes of three phyla (*Acidobacteria*, *Verrucomicrobia*, and *Gemmatimonadetes*), which are major soil phyla with many uncultured lineages (Janssen, 2006), revealed that several strains of *Acidobacteria* and *Verrucomicrobia* possessed D-CA homologs (Table S2). COS degrading activity of D-CA homologs from unknown soil majority has not yet been elucidated. This information will greatly contribute to understanding the distribution of COS-degrading activity in the D-CA family.

In conclusion, we herein developed a novel MPN method for enumerating COS degraders and found high densities of COS degraders in forest soil samples. This culture-dependent method enabled us to assign these degraders to *Bacillales* and *Actinomycetales*. Our results imply that these bacteria contribute to the uptake of atmospheric reduced sulfur by soils, and provide novel insights into the ecological roles of bacterial COS degradation in soils.

## Citation

Kato, H., Ogawa, T., Ohta, H., and Katayama, Y. (2020) Enumeration of Chemoorganotrophic Carbonyl Sulfide (COS)-degrading Microorganisms by the Most Probable Number Method. *Microbes Environ ***35**: ME19139.

https://doi.org/10.1264/jsme2.ME19139

## Supplementary Material

Supplementary Material

## Figures and Tables

**Table 1.** Parameters of soil and water samples.

Sampling site	Sample name	Sample type	Water content (%, w/wet wt)	pH (H_2_O)	TOC (%, w/dry wt)	Rate constant of COS degradation (h^–1^ [g dry soil]^–1^)
Mt. Karasawa	KS-13	Forest soil	29.7	5.28	4.8	1.10
Mt. Sengen-yama park	SG-1	Forest soil	31.0	5.0	5.2	1.25
Miyake-jima island	CL	Forest soil	46.1	5.4	7.0	1.25
	IG-C	Volcanic ash	25.3	4.1	<0.1	0.07
	IG-2A	Forest soil under ash	46.1	4.4	6.5	1.18
	OY-1-C	Volcanic ash	28.5	3.6	<0.1	0.01
	OY-1-A	Forest soil under ash	33.6	4.2	6.3	0.66
	KP-1	Scoria	10.2	4.9	<0.1	0.05
	KP-3	Scoria	17.8	4.9	0.38	0.22
	KP-5	Scoria	13.9	4.6	0.64	0.36
Mt. Fuji	F-1140*^)^	Forest soil	55.6	4.8	11.7	1.71
	F-1300	Forest soil	46.8	5.4	9.1	1.40
	F-1480	Scoria	10.5	5.4	0.29	0.60
	F-3020	Scoria	6.0	5.9	<0.1	0.27
	F-3230	Scoria	9.8	6.1	<0.1	0.27
	F-3590	Scoria	10.1	5.5	<0.1	0.35
	F-3750	Scoria	16.6	5.6	<0.1	0.26
Experimental pond	Pond A	Pond water		7.6	19.7	0.051
	Pond B	Pond water		7.7	11.3	0.035
	Pond C	Pond water		7.4	0.4	0.019

*) Numbers after “F-“ indicate the altitude at which samples were collected.

**Table 2.** Effects of the cultivation periods on the manifestation of COS-degrading activity in MPN cultures. Sample KS-13 collected at Mt. Karasawa was shown as an example. 1/10 NBY medium was used for the incubation of chemoorganotrophs.

Dilution level*	COS-degrading activity^†^			
	Incubation time from the inoculation (d)			
	4	11	18	40
2-1	**97**	**95**	**85**	**85**
2-2	**100**	**100**	**100**	**100**
2-3	**100**	**100**	**100**	**100**
3-1	**100**	**100**	**100**	**82**
3-2	**100**	**100**	**100**	**83**
3-3	**100**	**100**	**100**	**89**
4-1	**74**	**62**	**55**	**49**
4-2	**96**	**73**	**72**	**62**
4-3	**44**	**44**	**41**	34
5-1	**41**	**91**	**92**	**88**
5-2	**42**	**57**	**42**	37
5-3	38	**82**	**90**	**91**
6-1	22	**40**	**41**	37
6-2	23	**43**	**41**	31
6-3	28	**42**	**46**	39
7-1	**58**	**100**	**69**	**52**
7-2	25	**72**	**74**	—
7-3	22	29	24	**65**
8-1	24	29	27	29
8-2	23	28	**57**	39
8-3	23	26	26	26
9-1	25	28	26	28
9-2	25	27	26	26
9-3	24	27	26	**46**
10-1	25	27	30	27
10-2	25	26	26	27
10-3	26	26	26	27

*) For example, “2-1” means one of three replicates of the 10^2^ dilution of the soil sample from Mt. Karasawa. †) The degradation rate of 30 ppmv of COS for 24 h was assessed at each time point of the incubation. Numbers of positive cultures for COS degradation (≥40%) at three higher dilution levels (in this case: “3, 1, 1” from 10^7^ to 10^9^) gave a cell density of COS degraders of 9.6×10^8^ MPN (g dry soil)^–1^. Gray-colored cells indicate the heterotrophic growth of each MPN culture based on turbidity. The numbers of positive cultures for heterotrophic growth at three higher dilution levels (in this case: “3, 1, 1” from 10^7^ to 10^9^) gave a cell density of chemoorganotrophs of 9.6×10^8^ MPN (g dry soil)^–1^.

**Table 3.** Comparison of degrading activities among isolates obtained as COS-degrading bacteria.

Sample type	Source	Bacterial isolates	SRCB (h^–1^ mg^–1^ carbon)*
Forest soil	Aomori pref.	*Mycobacterium* sp. strain THI401^†^	2.6±0.1
	Mt. Karasawa	*Bacillus* sp. strain THI424	3.3±0.1
		*Bacillus* sp. strain THI419	2.6±0.2
		*Bacillus* sp. strain THI422	0.66±0.05
		*Streptomyces* sp. strain THI420	0.50±0.05
		*Bacillus* sp. strain THI418	0.46±0.04
		*Rhodococcus* sp. strain THI425	0.35±0.03
		*Streptomyces* sp. strain THI421	0.27±0.03
		*Bacillus* sp. strain THI417	0.034±0.008
		*Dyella* sp. strain THI423	0.030±0.001
Forest soil under ash	Miyake-jima island	*Bacillus* sp. strain THI427	2.3±0.1
		*Bacillus* sp. strain THI426	1.6±0.1
Volcanic ash	Miyake-jima island	*Bacillus* sp. strain THI428	2.3±0.04
		*Kitasatospora* sp. strain THI429	1.4±0.05
		*Lysinibacillus* sp. strain THI431	0.93±0.2
		*Lysinibacillus* sp. strain THI430	0.90±0.1

*) SRCB: Specific Rate Constant of COS degradation normalized by Biomass carbon. †) Details are described in Kato *et al.*, 2008.
