# Supplementary material for: Enumeration of Chemoorganotrophic Carbonyl Sulfide (COS)-degrading Microorganisms by the Most Probable Number Method
Source: Microbes Environ. 2020 Apr 29;35(2):ME19139. doi: 10.1264/jsme2.ME19139 (PMC7308577; doi:10.1264/jsme2.ME19139)
Supplement: Supplementary file 1 — Supplementary Material [file 35_19139_s1.pdf]

Supplementary table 1. Effect of the composition of the medium on MPN of COS degraders in soil samples of KS-13 and SG-1.

Soil sample KS-13

MPN medium		Number of MPN-positive cultures									Cell density
		10^{-2}	10^{-3}	10^{-4}	10^{-5}	10^{-6}	10^{-7}	10^{-8}	10^{-9}	10^{-10}	[MPN (g dry soil)$^{-1}$]
1/10 x NBY	chemoorganotrophs	3	3	3	3	3	3	1	1	0	$9.6x10^{8}$
	COS degraders	3	3	3	3	3	3	1	1	0	$9.6x10^{8}$
1/100 x NBY	chemoorganotrophs	3	3	3	3	3	3	0	0	0	$3.1x10^{8}$
	COS degraders	3	2	0 (2)*	1	0	1	0	0	0	$2.7x10^{4}$
1/2 x PYG	chemoorganotrophs	3	3	3	3	3	1	1	0	0	$9.6x10^{7}$
	COS degraders	3	3	3	3	3	1	1	0	0	$9.6x10^{7}$

Soil sample SG-1

MPN medium		Number of MPN-positive cultures									Cell density
		10^{-1}	10^{-2}	10^{-3}	10^{-4}	10^{-5}	10^{-6}	10^{-7}	10^{-8}	10^{-9}	[MPN (g dry soil)$^{-1}$]
1/10 x NBY	chemoorganotrophs	3	3	3	3	3	1	1	0	0	$1.1x10^{7}$
	COS degraders	3	3	3	3	3	0	0	0	0	$3.5x10^{6}$
Minimum medium with thiosulfate	Sulfur-oxidizing bacteria	3	3	3	3	0	0	0	0	0	$3.5x10^{5}$
	COS degraders	0	1	0	0	0	0	0	0	0	$4.3x10^{1}$

At each dilution level, numbers of MPN-positive cultures were counted based on COS degradation activity (≥40% of 30 ppmv COS was degraded in 24 hours) and microbial growth (turbidity of the culture). According to the reference of MPN table (Oblinger and Koburger, 1975), numbers at three dilution levels (gray-colored numbers) were selected to calculate MPN values. (*) The number in parentheses indicates the number of positives, in which positives from higher dilution levels (ones from 10^{-5} and 10^{-7}) were added.

Supplementary table 2. Blastp analysis of D-CA and COS-degrading enzymes against genomes of three major phyla in soil.
Blastp analysis (threshold: ≥ 40% identity) against the available genomes of *Acidobacteria, Verrucomicrobia, and Gemmatimonadetes* in Genbank database (https://www.ncbi.nlm.nih.gov)

Query : Carbonyl sulfide hydrolase (3VQJ_A) [*Thiobacillus thioparus* strain THI115]

identity (%)	e-value	bit score	Subject protein	Organisms	Conserved domains	Accession
40.0	2.38E-33	120	carbonic anhydrase	*Verrucomicrobium* sp. BvORR106	beta_CA_cladeD [cd03379]	WP_038165376.1

Query : Carbon disulfide hydrolase (G0WXL9) [*Acidianus* sp. strain A1-3]

identity (%)	e-value	bit score	Subject protein	Organisms	Conserved domains	Accession
no hit						

Query : Carbon disulfide hydrolase (AGQ48123) [*Acidithiobacillus thiooxidans* strain S1p]

identity (%)	e-value	bit score	Subject protein	Organisms	Conserved domains	Accession
no hit						

Query : Carbon disulfide hydrolase (AGQ48122) [*Acidithiobacillus thiooxidans* strain G8]

identity (%)	e-value	bit score	Subject protein	Organisms	Conserved domains	Accession
no hit						

Query : Clade D type of beta carbonic anhydrase (NP_215800) [*Mycobacterium tuberculosis* strain H37Rv]

identity (%)	e-value	bit score	Subject protein	Organisms	Conserved domains	Accession
41.7	3.88E-41	138	carbonic anhydrase	*Verrucomicrobiae* bacterium DG1235	beta_CA_cladeD [cd03379]	WP_008102208.1
41.3	5.92E-39	132	carbonic anhydrase	*Methylacidiphilum infernorum*	beta_CA_cladeD [cd03379]	WP_012464703.1
40.6	8.53E-39	132	carbonic anhydrase	*Methylacidiphilum kamchatkense*	beta_CA_cladeD [cd03379]	WP_039721106.1
40.0	1.96E-37	129	carbonic anhydrase	*Methylacidiphilum fumariolicum*	beta_CA_cladeD [cd03379]	WP_009061375.1
40.9	2.14E-36	126	carbonic anhydrase	*Verrucomicrobia* bacterium LP2A	beta_CA_cladeD [cd03379]	WP_024808582.1
40.0	2.54E-40	135	hypothetical protein	*Acidobacteria* bacterium 13_2_20CM_2_57_6	beta_CA_cladeD [cd03379]	OLB85114.1
41.2	7E-40	134	hypothetical protein	*Acidobacteria* bacterium 13_1_20CM_58_21	beta_CA_cladeD [cd03379]	OLD81336.1
40.0	1.52E-39	133	hypothetical protein	*Acidobacteria* bacterium 13_2_20CM_57_17	beta_CA_cladeD [cd03379]	OLB39356.1
40.1	4.29E-39	133	hypothetical protein	*Acidobacteriaceae* bacterium KBS 96	beta_CA_cladeD [cd03379]	WP_020722144.1
40.3	2.9E-36	126	carbonic anhydrase	*Edaphobacter aggregans*	beta_CA_cladeD [cd03379]	WP_035357967.1
